# Complete genome sequence of *Chelatococcus* sp. strain KU-N122 (JCM 37729) isolated from a cyclooctanone enrichment culture

**DOI:** 10.1128/mra.01021-25

**Published:** 2025-10-31

**Authors:** Taisei Yamamoto, Kenji Okano, Hiroaki Iwaki

**Affiliations:** 1 Department of Life Science & Biotechnology, Kansai University 12860, Suita, Japan; University of Southern California, Los Angeles, California, USA

**Keywords:** *Chelatococcus*, cyclooctanone enrichment, complete genome sequence, Baeyer–Villiger monooxygenase, cycloalkanone monooxygenase

## Abstract

Here, we report the complete genome sequence of *Chelatococcus* sp. strain KU-N122, isolated from a cyclooctanone enrichment culture. The genome comprises a single circular chromosome containing 7,983 protein-coding sequences. The strain was deposited in the Japan Collection of Microorganisms under the accession number JCM 37729.

## ANNOUNCEMENT

The cyclooctanone degradation pathway is valuable in synthetic organic chemistry, primarily because cycloalkanone monooxygenase (CAMO), a Baeyer–Villiger monooxygenase (BVMO), catalyzes its initial step (1). This study reports the complete genome sequence of strain KU-N122, isolated from a cyclooctanone enrichment culture, and provides insights into cyclooctanone metabolism and putative BVMO-encoding genes.

Strain KU-N122 was isolated from roadside soil in Sagamihara, Kanagawa, Japan (latitude: 35.57; longitude: 139.41). Soil (0.1 g) was inoculated into a mineral salts medium (5 mL) (2) containing 0.1% cyclooctanone as the sole carbon source and incubated at 120 rpm and 30°C. After 3 days, the culture (25 µL) was transferred to fresh medium and incubated for another 3 days; this process was repeated once. The strain was purified as described previously (3) and deposited in the Japan Collection of Microorganisms under accession number JCM 37729.

Genomic DNA was extracted from a 50 mL LB (Miller) culture grown for 24 h at 30°C using sodium dodecyl sulfate/proteinase K lysis (after resuspension in Tris-EDTA buffer [15 mL] containing RNase A; subsequent reagent volumes were scaled to this volume) and phenol–chloroform extraction (4). To prepare the 20 kb SMRTbell library, genomic DNA (8 µg) was sheared into 20 kb fragments as described previously (5). Purification with AMPure PB magnetic beads (Beckman Coulter) was conducted to remove short fragments. A 20 kb SMRTbell template library was prepared using the SMRTbell Template Prep Kit (Pacific Biosciences) and sequenced using a PacBio RS II instrument. Subreads were filtered as previously described (5) with minimum subread and polymerase read lengths of 500 and 100 bp, respectively, and a minimum polymerase read quality of 0.80. Default parameters were used for all tools unless otherwise noted. In total, 112,389 reads (1,212,535,341 bp, with an *N*_50_ value of 16,507 bp) were obtained. The reads were assembled using the Hierarchical Genome Assembly Process (HGAP) version 3 (6), resulting in one circular contig with 101× coverage. HGAP automatically identifies and trims the overlapping ends, completing genome circularization. The complete genome sequence of strain KU-N122 consisted of an 8,825,821 bp circular chromosome with a GC content of 68.02%.

The genome sequence was annotated using DFAST (v.1.6.0) (7), identifying 7,983 coding sequences, 63 tRNA genes, and 9 rRNA genes. A BLASTP search using *in silico* Molecular Cloning software (*in silico* Biology) with a CAMO (LC610772) as the query identified 11 putative BVMO genes. Among the putative BVMOs, KUN122_12700 was the most similar (50.4% identity) and was identified as a candidate CAMO.

Strain KU-N122 was identified based on 16S rRNA gene sequencing and digital DNA–DNA hybridization (dDDH) (8, 9). The 16S rRNA gene sequence similarity was analyzed using BLASTN (10), and the dDDH value was calculated using the DSMZ Type (strain) Genome Server (11). The 16S rRNA gene sequence showed the highest similarity (99.03%) to *Chelatococcus reniformis* (NR_152704). Similarly, the highest dDDH value (formula *d*_4_) was recorded for *C. reniformis*, but it was only 31.6%. Based on the genomic criteria (11), strain KU-N122 is considered a putatively novel species within the genus *Chelatococcus*.

## Data Availability

The genome sequence is available from DDBJ/EMBL/GenBank with accession number AP043981. The associated BioProject, BioSample, and Sequence Read Archive accession numbers are PRJDB16010, SAMD00623165, and DRR483476, respectively.
